# Characterization of Chicken IgY Specific to *Clostridium difficile* R20291 Spores and the Effect of Oral Administration in Mouse Models of Initiation and Recurrent Disease

**DOI:** 10.3389/fcimb.2017.00365

**Published:** 2017-08-14

**Authors:** Marjorie Pizarro-Guajardo, Fernando Díaz-González, Manuel Álvarez-Lobos, Daniel Paredes-Sabja

**Affiliations:** ^1^Microbiota-Host Interactions and Clostridia Research Group, Departamento de Ciencias Biologicas, Universidad Andres Bello Santiago, Chile; ^2^Departamento de Gastroenterología, Facultad de Medicina, Pontificia Universidad Católica Santiago, Chile

**Keywords:** *Clostridium difficile* spores, chicken immunoglobulin IgY, immunotherapy, passive immunization, recurrent CDI, exosporium, germination, sporulation

## Abstract

*Clostridium difficile* infection (CDI) are the leading cause of world-wide nosocomial acquired diarrhea. The current main clinical challenge in CDI is the elevated rate of infection recurrence that may reach up to 30% of the patients, which has been associated to the formation of dormant spores during the infection. We sought to characterize the effects of oral administration of specific anti-spore IgY in mouse models of CDI and recurrent CDI. The specificity of anti-spore IgY was evaluated *in vitro*. In both, initiation mouse model and recurrence mouse model, we evaluated the prophylactic and therapeutic effect of anti-spore IgY, respectively. Our results demonstrate that anti-spore IgY exhibited high specificity and titers against *C. difficile* spores and reduced spore adherence to intestinal cells *in vitro*. Administration of anti-spore IgY to C57BL/6 mice prior and during CDI delayed the appearance of the diarrhea by 1.5 day, and spore adherence to the colonic mucosa by 90%. Notably, in the recurrence model, co-administration of anti-spore IgY coupled with vancomycin delayed the appearance of recurrent diarrhea by a median of 2 days. Collectively, these observations suggest that anti-spore IgY antibodies may be used as a novel prophylactic treatment for CDI, or in combination with antibiotics to treat CDI and prevent recurrence of the infection.

## Introduction

*Clostridium difficile* is a spore-forming Gram-positive anaerobic bacterium and is the most frequently identified cause of nosocomial diarrhea. It has been associated with epidemics of diarrhea in hospitals and long-term care facilities (Cohen et al., 2010; Evans and Safdar, 2015). Symptoms associated with *C. difficile* infections (CDI) range from asymptomatic colonization to severe diarrhea, pseudomembranous colitis, toxic megacolon, colonic perforation and even death (Gerding et al., 1995). A narrow set of antibiotics (i.e., metronidazole and vancomycin) are currently being used to treat CDI (Evans and Safdar, 2015); albeit these antibiotics resolve the first episode of the disease, the rate of infection recurrence can be as high as 30% (Bakken et al., 2013).

Unlike most enteropathogens, during the infection *C. difficile* initiates a sporulation cycle that culminates with the production of metabolically dormant spores are shedded to the environment causing disease persistence and transmission (Deakin et al., 2012); these spores survive inside cause recurrence of the infection. Given the natural resistance of *C. difficile* spores to antibiotics, they remain in dormant state and may germinate upon cease of antibiotic treatment, generating a new infection (Rupnik et al., 2009). New spore-aimed therapies targeting the removal of *C. difficile* spores from a susceptible host and during CDI may prevent the initiation and recurrence of the disease, respectively. Consequently, the opsonization of *C. difficile* spores with spore-specific antibodies may be conceived as a possible strategy to reduce *C. difficile* spore-host interactions and *C. difficile* spore-load in the host.

Chicken IgY has several advantages over other immunoglobulins for the development of passive immunotherapy: (i) current regulations of the Federal Drug Administration (FDA) classifies the oral consumption of IgY as “Generally Recognized as Safe” (GRAS), facilitating the regulations for human consumption of pathogen-specific IgY (Rahman et al., 2013); (ii) oral administration of IgY does not trigger an adaptive immune response against administered immunoglobulin (Nilsson et al., 2007); (iii) the structure of IgY is not recognized by intestinal epithelial Fc-receptors in mammals and does not activate mammalian complement (Carlander et al., 2000; Kovacs-Nolan and Mine, 2012); (iv) IgY exhibits high antigen-specificity and avidity (Kovacs-Nolan and Mine, 2012), and faster reaction times with the antigen than mammalian IgG (Rahman et al., 2013); and (v) IgY retains their activity during transit through the gastrointestinal tract (Kovacs-Nolan and Mine, 2012). Notably, attempts to use passive immunization to treat CDI have focused on both toxins, TcdA and TcdB, and on structural components of vegetative cells (Mulvey et al., 2011; Zhang S. et al., 2015). However, anti-spore IgY have not been explored.

Consequently, the aim of this work was to raise and characterize anti-spore IgY and to evaluate if its oral administration could protect mice from the initiation and recurrence of the infection. In this work, we demonstrate the feasibility of *C. difficile* spore-targeted therapies to prevent the initiation and recurrence of CDI.

## Materials and methods

### Clostridium difficile strains

*C. difficile* strain R20291 (RT027) is an epidemic strain that caused outbreaks and has been described elsewhere (McEllistrem et al., 2005; He et al., 2010). *C. difficile* clinical isolates PUC31 (RT046), PUC38 (RT082) and PUC104 (RT097) were obtained from Chilean patients and have been described elsewhere (Plaza-Garrido et al., 2015). *C. difficile* strains were grown in a Bactron II-2 anaerobic chamber (Shellab, OR, U.S.A.) in 3.7% of Brain Heart Infusion supplemented with 0.5% yeast extract (BHIS) broth or on BHIS agar plates.

### Spore purification

*C. difficile* spores were purified as described elsewhere (Mora-Uribe et al., 2016). Spore suspensions were prepared by plating a 1:500 dilution from an overnight culture onto 3% Trypticase Soy-0.5% yeast extract (TY) agar plates and incubated for 5 days at 37°C under anaerobic conditions. Spores were harvested with ice-cold sterile distilled water and purified with 50% Nicodenz as previously described (Sorg and Sonenshein, 2008). Spore suspensions were purified until they were >99% free of vegetative cells, sporulating cells and cell debris as determined by phase contrast microscopy and the concentration was quantified with a Neubauer Chamber (Sigma-Aldrich, U.S.A.) prior to use.

### Chicken immunization protocol

Chicken immunoglobulins (IgY) specific for *C. difficile* spores from the epidemic strain R20291 were produced as described by the manufacturer (AvesLabs, OR, U.S.A.). Briefly, 5 × 10^9^ R20291 spores were fixed in 4% paraformaldehyde for 16 h at 4°C, rinsed with PBS, and aliquots of 500 μL PBS containing 2.5 × 10^8^ spores were stored at −20°C until use. Two hens were immunized intramuscularly at four separate locations of the hen's breast tissue (250 μL per site) with a 1 ml solution containing 500 μL antigen mixed 1:1 with Freund's complete adjuvant. After weeks 3, 5, and 7, booster shoots of 500 μL antigen mixed 1:1 with incomplete Freund's (50%) adjuvant were administered. Eggs were collected from weeks 7 to 11 and were used for IgY purification of each different hen.

### Purification of anti-*C. difficile* spore IgY

Two independent batches of chicken immunoglobulins were obtained as pure IgY from AvesLab as described by manufacturer using described protocols (Akita and Nakai, 1992, 1993; Liou et al., 2010). However, since the IgY suspension had a IgY(ΔFc) isotype which lacks the Fc region (Lundqvist et al., 2006), a further purification step was applied to obtain purified full length IgY fraction by salting out precipitation under acidic conditions, as described (Hodek et al., 2013). Briefly, AvesLab IgY suspension was diluted in 20 volumes of 8.8% NaCl w/v, adjusted at pH 4.0 with HCl and incubated with shaking for 2 h at room temperature. The precipitated IgY was collected by centrifugation (3,700 g for 20 min) at 4°C and the pellet resuspended in 1 volume of PBS, filtered sterilized, and stored at −20°C until use. The purity of purified IgY was analyzed by SDS-PAGE and Western blot analysis using anti-IgY donkey IgG conjugated with HRP (Abcam, U.S.A.).

### Enzyme-linked immunosorbent assay of antibody titer against *C. difficile* spores of the epidemic strain R20291

Antibody titers were assessed by enzyme-linked immunosorbent assay (ELISA) using previously described protocols (Snyder et al., 1983). Briefly, 96-well ELISA plates (Grainer, Germany) were incubated for 16 h at 4°C with 100 μL of PBS containing 1.6 × 10^7^ spores of strain R20291. Unbound spores were removed by washing the well three times with 150 μL of PBS. Bounded spores were further blocked with 2% BSA in PBS-0.05% Tween 20 (PBS-T) for 1 h at 37°C, rinsed 5 times with 150 μl PBS-T. Wells were next incubated with serial dilutions of anti-spore IgY in 1% BSA in PBS-T for 2 h at 37°C, rinsed five times with 150 μL of PBS-T, and further incubated with a 1:10,000 dilution of anti-chicken IgY-horseradish peroxidase conjugate (Anti-IgY HRP) (Abcam, U.S.A.) in 1% BSA in PBS-T for 1 h at 37°C. Wells were finally rinsed five times with 150 μL of PBS-T. Colorimetric reaction was initiated upon addition of 50 μL of reaction buffer (0.05 M citric acid, 0.1 M disodium hydrogen phosphate) containing 2 mg/mL of o-phenlyendiamine (Sigma-Aldrich, U.S.A.) and 0.015% of H_2_O_2_ (Merck, Germany). Reaction was stopped after 20 min with 25 μL of 4.5 N of H_2_SO_4_ and absorbance was measured at 492 nm. Background reactivity was performed using IgY from eggs obtained prior immunization. All experiments were repeated three times in triplicate. Antibody titers were analyzed as previously described (Snyder et al., 1983).

### Immunofluorescence

Spores were fixed on poly-L-lysine-coated glass cover slides with 3% paraformaldehyde (pH 7.4) for 20 min. Next, fixed spores were rinsed three times with PBS, blocked with 1% bovine serum albumin (BSA) for 1 h and further incubated for 1 h at room temperature with 1:100 of anti-spore IgY. Covers were rinsed three times with PBS and incubated for 1 h at room temperature with 1:500 goat anti-IgY conjugated with Alexa 488 (Invitrogen) in PBS with 1% BSA and washed three times with PBS and once with sterile distilled water. Samples were then dried at room temperature for 30 min, and covers were mounted using Dako Fluorescence Mounting medium (Dako, North America) and sealed with nail polish. Samples were analyzed in an Olympus BX53 fluorescence microscope. Control experiments included spores without primary antibodies, which did not yield fluorescence signal (data not shown). Using ImageJ (v1.48, NIH), an outline was drawn around 300 spores for experimental condition (*n* = 100 per replicate) including the integrated density and the mean fluorescence, along with several adjacent background readings.

### SDS-PAGE and western immunoblotting of *C. difficile* vegetative cells, spores and murine fecal bacterial cells

Samples (10 μL) containing 4 × 10^7^ cells or spores of *C. difficile* strain R20291, or similar number of cells of murine microbiota bacterial cells in SDS-PAGE loading buffer, were electrophoresed on SDS-PAGE gels (12% acrylamide). Proteins were transferred to a nitrocellulose membrane (Bio-Rad) and blocked for 1 h at room temperature with 2% BSA-Tris buffered saline pH 7.4 (TBS). These Western blots were probed with a 1:5,000 dilution of anti-spore IgY in 2% BSA-TBS-0.1% Tween 20 (2% BSA-TTBS) for 2 h at room temperature, rinsed three times with TTBS and incubated a 1:10,000 rabbit anti-IgY HRP in TTBS for 1 h at room temperature. Membranes were rinsed three times with TTBS and a final wash with TBS. HRP activity was detected with a chemiluminescence detection system (Fotodyne Imaging) by using Pixo*Max* sensitive chemiluminescent detection system HRP-substrate (RockLand Immunochemicals).

### ELISA of anti-spore IgY against *C. difficile* vegetative cells and murine fecal microbiota

Murine fecal microbiota was obtained from untreated and antibiotic treated mice. Feces (250 mg) were hydrated in 10 ml PBS at 4°C for 16 h with gentle stirring. Fecal macroscopic organic material was removed by low speed centrifugation (500 rpm; 27 × g) at 4°C for 5 min, this step was repeated four times and cells were resuspended to a final concentration of 1.6 × 10^8^ cell/mL. To evaluate the immunoreactivity of IgYs with conformational antigens, 96-well ELISA plates (Grainer, Germany) were incubated for 16 h at 4°C with 100 μL of PBS containing 4 × 10^7^ fecal microbiota bacterial cells, spores or vegetative cells of *C. difficile*. Unbound cells/spores were removed by washing the well three times with 150 μL of PBS and antibody titers were analyzed as previously described (Snyder et al., 1983).

### Germination assay

*C. difficile* spores were pre-incubated for 2 h at 37°C with various concentrations of anti-spore IgY, and unbound IgY was removed by three washes. IgY-treated and untreated spores were serially diluted and plated onto BHIS-ST agar plates and incubated anaerobically at 37°C for 36 h.

### Adherence assay

Caco-2 cells were seeded (5 × 10^5^ cells per well) onto 96-well plates and incubated until cells became confluent, and further incubated for 8 days, obtaining differentiated Caco-2 monolayers. Differentiation was assessed by immunofluorescence using sucrose-isomaltase as a marker for the appearance of microvilli (Mora-Uribe et al., 2016). Before infection, *C. difficile* spores were pre-incubated for 2 h at 37°C with various concentrations of anti-spore IgY, and unbound IgY was removed by three washes with PBS. Pre-incubated spores were used to infect monolayers of differentiated Caco-2 cells at a multiplicity of infection (MOI) of 10 for 3 h at 37°C and 5% CO_2_ atmosphere. Unbound spores were removed by 3 washes with PBS. Caco-2 cells were then lysed with 95 μl 0.06% Triton X-100 for 30 min at 37°C, and appropriate dilutions, containing adhered spores, were plated onto BHIS-ST agar plates and incubated under anaerobic conditions at 37°C for 36 h. For total *C. difficile* spore counts, infected Caco-2 cells were directly lysed and plated onto BHIS-ST agar and incubated anaerobically at 37°C for 36 h. Numbers of colony forming units (CFU) were determined and percentages of adherence were calculated using the following formula: ([final CFU/mL]/[initial CFU/mL]) × 100; where initial CFU correspond to colonies from unwashed wells. These experimental conditions do not trigger germination of *C. difficile* spores (Paredes-Sabja and Sarker, 2011).

### Mice

Pathogen-free C57BL/6 mice (age 6–9 weeks) were obtained from the Institute of Public Health of Chile (Santiago, Chile). All mice used in the experiments were housed in groups of 4% under conventional conditions at the Animal Infection Facility of the Microbiota-Host Interactions and Clostridia Research Group of Universidad Andrés Bello (Santiago, Chile), for 2 weeks before the initiation of the study. Food (Chow; Prolab RMH 3000 rodent diet, St. Louis, MO, U.S.A), water, bedding and cages were sterilized prior use. All experimental protocols were conducted in strict accordance with, and under the formal approval of the Institutional Animal Ethics Committee of the Universidad Andrés Bello.

### Animal infection model

Prior to infection, mice were pre-treated with antibiotic cocktail of kanamycin (4.2 mg/kg body weight; Sigma-Aldrich, U.S.A.), gentamicin (3.5 mg/kg body weight; Sigma-Aldrich), colistin (4.2 mg/kg body weight; Sigma-Aldrich), metronidazole (21.5 mg/kg body weight; Sigma-Aldrich) and vancomycin (4.5 mg/kg body weight; Sigma-Aldrich) for 3 days, in the drinking water (Chen et al., 2008). After the treatment, mice received sterile water for 2 days, followed by intraperitoneal administration of a single dose of clindamycin (10 mg/kg) 1 day before *C. difficile* challenge (Chen et al., 2008). All animals were infected orogastrically with 200 μl PBS containing 6 × 10^7^ spores of strain R20291. Mice were housed individually in sterile cages with *ad libitum* access to food and water. All procedures and mouse handling were performed aseptically in a biosafety cabinet to contain spore-mediated transmission.

Preliminary evaluation of dose efficacy includes anti-spore IgY administration prior to infection of mice (*n* = 4 per treatment), consisting in orogastric administration of a first dose of 50 μl of PBS containing 100 and 200 μg of anti-spore IgY or 200 μg of pre-immunized IgY 2 h prior to infection by oral gavage, a second dose 6 h post-infection and thereafter doses every 24 h during the following 3 days.

To evaluate the effect of orogastric administration of a higher dose of anti-spore IgY prior to infection of mice (*n* = 12 per treatment), a first dose of 50 μl of PBS containing 600 μg anti-spore IgY or PBS was given by oral gavage 2 h prior to infection. A second dose was given 6 h post-infection, and thereafter doses where given every 24 h during the following 3 days.

To evaluate the effect of anti-spore IgY in the prevention of recurrence of CDI, two groups of mice (*n* = 10) were pre-treated with antibiotics and infected with *C. difficile* as described above. From days 3 to 9, mice were orally administered with 50 μl of PBS containing vancomycin (50 mg/kg; Sigma-Aldrich) or a combination of vancomycin (50 mg/kg) and anti-spore IgY (600 μg/dose).

The clinical condition of mice was monitored daily with a scoring system. The presence of diarrhea was classified according to severity as follows: (i) normal stool (score = 1); (ii) color change/consistency (score = 2); (iii) presence of wet tail or mucosa (score = 3); (iv) liquid stools (score = 4). A score higher than 1 was considered as diarrhea (Warren et al., 2013). Other clinical symptoms analyzed were weight, physical aspect (i.e., abnormal/hunched gait, piloerection), spontaneous behavior (i.e., lethargy, inactivity or lack of mobility) and emaciation were monitored as described (Deakin et al., 2012). Moribund mice or mice displaying overt signs of disease should sacrificed.

### Quantification of spores from feces and colon

Fecal samples were collected daily and 40 mg of feces were hydrated with 500 μl in sterile MilliQ water for 16 h at 4°C, and then added 500 μL of absolute ethanol (Sigma-Aldrich) which were incubated for 60 min at room temperature. Samples were serially diluted and plated onto selective medium supplemented with taurocholate (0.1% w/v), Cefoxitin (16 μg/mL), L-cycloserine (250 μg/mL) (TCCFA plates). The plates were incubated anaerobically at 37°C for 48 h, colonies counted and results expressed as the Log10 [CFU/gram of feces].

Colonic tissue was collected from mice, washed three times with PBS with a syringe. The spore load in the colon was determined using the first centimeter (cm) of the proximal region of the colon measured from the base of the cecum; ~70 mg of tissue was obtained, hydrated with 700 μL of PBS and homogenized. The amounts of spores were quantified by heat treating the sample at 65°C to kill vegetative cells and plating onto TCCFA plates. The plates were incubated anaerobically at 37°C for 48 h. Finally, the colony count was expressed as the Log10 [CFU/gram of colon].

### IgG reactivity assay

Blood from of infected mice were collected by cardiac puncture. After 30 min at 37°C, sample was centrifuged at 4,000 rpm for 20 min at 4°C and the supernatant, containing the serum fraction was stored at −20 until use. To assess the reactivity of serum IgG against *C. difficile* spores and IgY from treatment, ELISA assay was performed in wells incubated with R20291 spores (1.6 × 10^7^ spores) or IgY (100 μg/mL), respectively. After blocking and 3 washes with 100 μL PBS, mouse serum was added at 1/500, 1/1,000, and 1/2,000 dilution and incubated for 2 h at 37°C. After that, non-adherent IgG was removed from the wells with 5 washes with PBS-T, followed by incubation with secondary antibody, anti-mouse HRP, for 1 h at 37°C. Finally, colorimetric reaction was developed as described before.

### Cecum content cytotoxicity assay

Vero cell cytotoxicity was performed as described previously (Theriot et al., 2011). Briefly, Vero cells were seeded in a 96-well flat bottom microtiter plate at a density of 10^5^ cells/well. Cecum contents from mice were suspended in PBS at a ratio of 1:10 (10 μl of PBS per mg of cecum content), vortexed and centrifuged (14,000 rpm, 5 min). Supernatant was filter sterilized and serially diluted in DMEM supplemented with 10% FBS and 1% penicillium streptomycin and 100 μl of each dilution was added to Vero cell wells. After 16 h of incubation at 37°C, plates were screened for cell rounding. The cytotoxic titer was defined as the reciprocal of the highest dilution that produced rounding in at least 80% of Vero cells per gram of luminal samples under X200 magnification.

### Histopathology evaluation

Light microscopy evaluation was performed by a veterinary pathologist in a blinded manner (unaware of the time points and experimental design at the time of evaluation). Histological lesions were categorically scored from 0 (normal) to 4 (most severe) for edema, inflammation, and epithelial damage using a scoring system reported previously (Kelly et al., 1994). Individual parameter scores were added for a summary of the histological score with a maximum value of 12. Samples from uninfected mice included as internal negative controls received a score of zero using the same grading scale and assessed by the same pathologist. Representative photomicrographs were taken using a MicroPublisher 3.3 megapixels digital camera mounted to an Olympus BX53 light microscope.

### Statistical analysis

Prism 6 (GraphPad Software, Inc.) was used for statistical analysis. Significance between groups was done by Mann-Whitney unpaired *t*-test. Comparative analysis between groups for *in vitro* experiments was analyzed by analysis of variance with *post-hoc* Student *t*-tests with Bonferroni corrections for multiple comparison, as appropriate. A *P*-value of ≤0.05 was accepted as the level of statistical significance. Differences in the percentages of mice with normal stools, as well as percentages of mice with *C. difficile* infection were determined with by Gehan-Breslow-Wilcoxon test.

## Results

### Purification of full length chicken anti-spore IgY

Previous studies of passive immunotherapies against *C. difficile* infections have aimed to neutralize toxins A and B (Zhang Z. et al., 2015), flagella or surface layer proteins (Mulvey et al., 2011). In the present study, we aim to evaluate whether oral administration of anti-spore IgY could prevent the initiation of the infection in a mouse model when administered alone, or the recurrence of the infection when co-administered with vancomycin. Given the emergence of *C. difficile* NAP1/BI/027 strains, including strain R20291, which has caused epidemics of CDI (O'Connor et al., 2009; He et al., 2013), we selected this strain to immunize hens. In addition, this strain is a good sporulating strain *in vitro*, and the mouse models of initiation and recurrence for this strain have been well characterized (Deakin et al., 2012; Winston et al., 2016).

**Figure 1:**
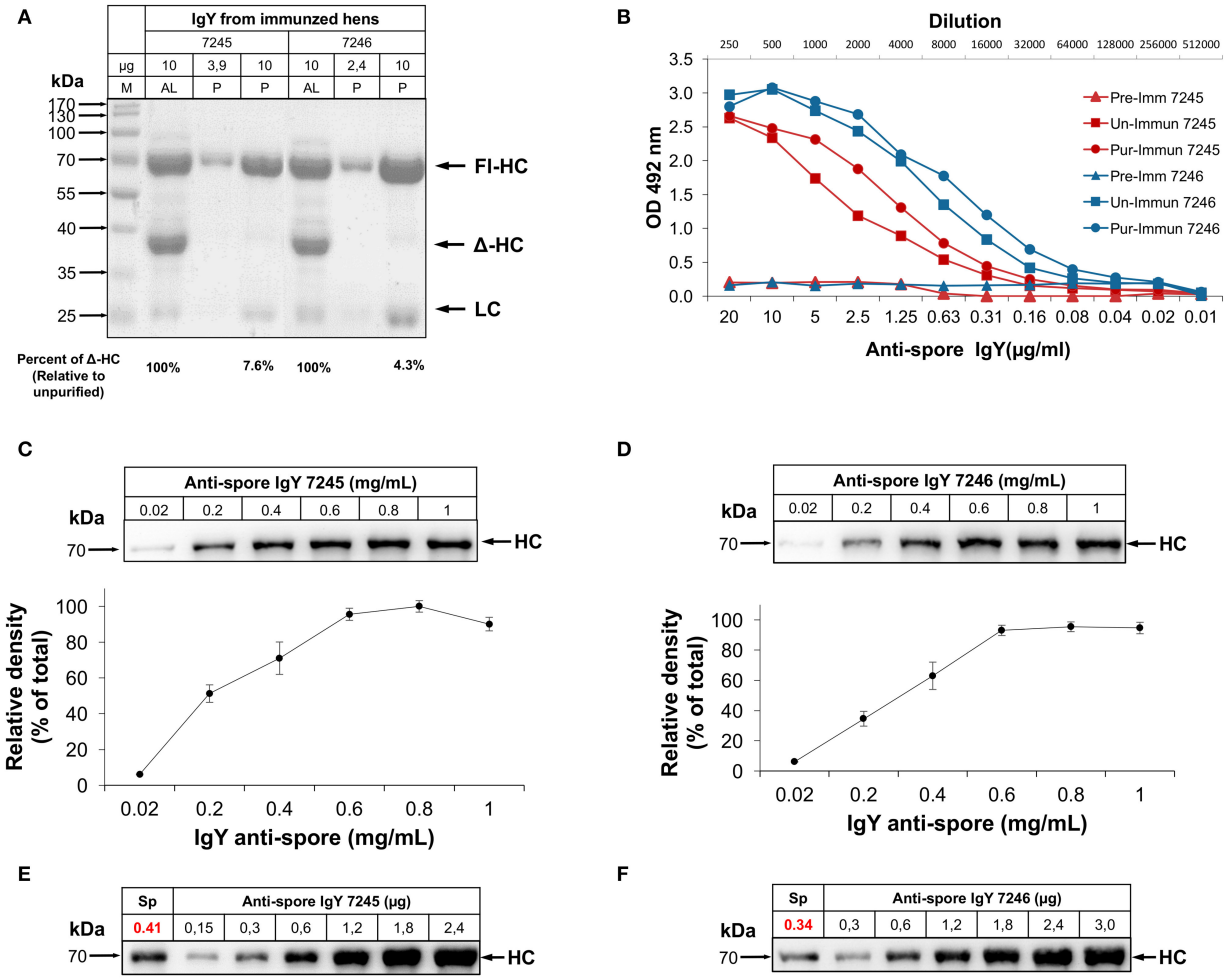
Characterization of anti-spore IgY specificity against *C. difficile* spores. **(A)** SDS-PAGE of the purification of full length IgY (P) from IgY obtained from the manufacturer (AL). AL, IgY purified in Aves Labs; P, IgY purified. Arrows show band corresponding to heavy chain (HC), light chain (LC) of IgY and HC-Δ corresponding to alternative splicing. **(B)** Titers of purified anti-spore IgY (Pur) and unpurified anti-spore IgY (Un) of batches 7245 and 7246 and their corresponding preimmunized IgY (Pre) were compared by ELISA. **(C,D)** Saturation concentration of IgY binding to *C. difficile* spores. 4 × 10^7^ spores were incubated with different concentrations of anti-spore IgY of batches 7245 **(C)** and 7246 **(D)** and the amount of the heavy chain IgY (HC) bound to the spore surface was determined by Western blot with anti-IgY HRP (Abcam). Densitometry analysis of the amount of IgY bounded to the spore surface was analyzed with ImageJ and expressed as relative to saturation levels. **(E,F)** Quantitation of the IgY level bound to *C. difficile* spores. 4 × 10^7^ spores were incubated with anti-spore IgY of batches 7245 and 7246 at saturation concentration (1.2 mg/ml), rinsed and the amount of IgY bound to the spore surface determined by a calibration curve of IgY, and estimated to be 0.34 μg of IgY per 4 × 10^7^ spores.

SDS-PAGE analysis of IgY suspensions of both batches of anti-spore IgY (7245 and 7246) obtained from AvesLab, revealed the presence of the three dominant bands, a 67-70-kDa molecular size species that represents the heavy “nu” chain (Fl-HC), and a 22-kDa molecular size species corresponding to the light chain (LC) (Figure S1A). A third band was detected with an estimated molecular size of 40-kDa (Figure S1A), which corresponds to a smaller form of the heavy chain of IgY (Δ-HC), produced by some galliform and non-galliform birds (Lundqvist et al., 2006; Li et al., 2012). These bands were also observed in electrophoresed egg yolk from locally acquired eggs (Figure S1A). Western blot analysis with polyclonal goat IgG against IgY revealed the presence of two main immunoreactive bands with similar molecular sizes as Fl-HC and LC, indicating that the Δ-HC is not recognized by the polyclonal anti-IgY antibody (Figure S1B). To remove the IgY (ΔFc) fraction, full-length IgY was further purified under acidified (pH 4.0) conditions (Hodek et al., 2013) to a purity of 98% (Figure S1C). Analysis of the purified fractions of anti-spore IgY revealed an increase in purity of 92 and 95%, for batches 7245 and 7246, respectively (Figure 1A). Acidic precipitation of IgY does not affect the activity of the antibody, as the immunoglobulins present high titer against spores. The titers of purified fractions of anti-spore IgY (i.e., reciprocal of antibody dilution) for both IgY fractions were determined by ELISA. The antibody titers for the unpurified IgY fractions of batches 7245 and 7246 were estimated in 1:128,000 and 1:256,000, respectively; after purification of full-length IgY, titers of batches 7245 and 7246 increased to 1:256,000 and 1:512,000, respectively (Figure 1B). These results indicate that acidic purification of full-length IgY did not affect anti-spore IgY titers against *C. difficile* spores.

### Saturation and level of binding of anti-spore IgY to *C. difficile* spores

Nearly 2–10% of IgY has been reported to be antigen specific (Kovacs-Nolan and Mine, 2004). Therefore, to gain more information on the anti-spore IgY we determined the percentage of IgY specific to *C. difficile* spores to optimize downstream therapeutic development. The saturation point of the anti-spore IgY required to cover the surface of 4 × 10^7^ spores was determined by pre-incubating *C. difficile* spores with 0.2 to 1.0 mg/mL of anti-spore IgY from batches 7245 and 7246 followed by SDS-PAGE electrophoresis and immunoblotting (Figures 1C,D). Figures 1C,D demonstrates that *C. difficile* spores become saturated with a concentration of anti-spore IgY of 0.6 mg/mL from either of both batches (i.e., 7245 and 7246). To determine the number of molecules bound to the spore surface, a fix number of *C. difficile* spores was incubated with a fixed amount of IgY of batches 7245 and 7246. After washes to remove non-adhered antibodies, proteins from the spores and attached antibodies were resolved by SDS-PAGE against a gradient of IgY, and immunoblotted with anti-IgY antibody. Results show that 0.41 and 0.34 μg of IgY from batches 7245 and 7246, respectively, were estimated to bind to 4 × 10^7^ spores by densitometry (Figures 1E,F). This corresponds to ~1% of total IgY (example of calculation for batch 7246: [percentage of IgY = (0.34 μg/(0.6 μg/μL) × 50 μL) × 100]). The number of anti-spore IgY molecules per *C. difficile* spore of batches 7245 and 7246 were estimated to be 87,141 and 73,124, respectively (example of calculation for batch 7246: [Number of IgY per spores = (0.34 μg/((70,000 Da/(6,022 × 10^23^) × 10^6^/(4 × 10^7^]) (numbers are representative of three independent experiments with essentially similar results); indicating an adequate level of opsonization of the spore surface with anti-spore IgY. Based on these results we designed downstream experiments (i.e., effect on spore adherence, germination and *in vivo* infection) using saturation concentration.

### Immunoreactivity of anti-spore IgY against *C. difficile* vegetative cells and spores

**Figure 2:**
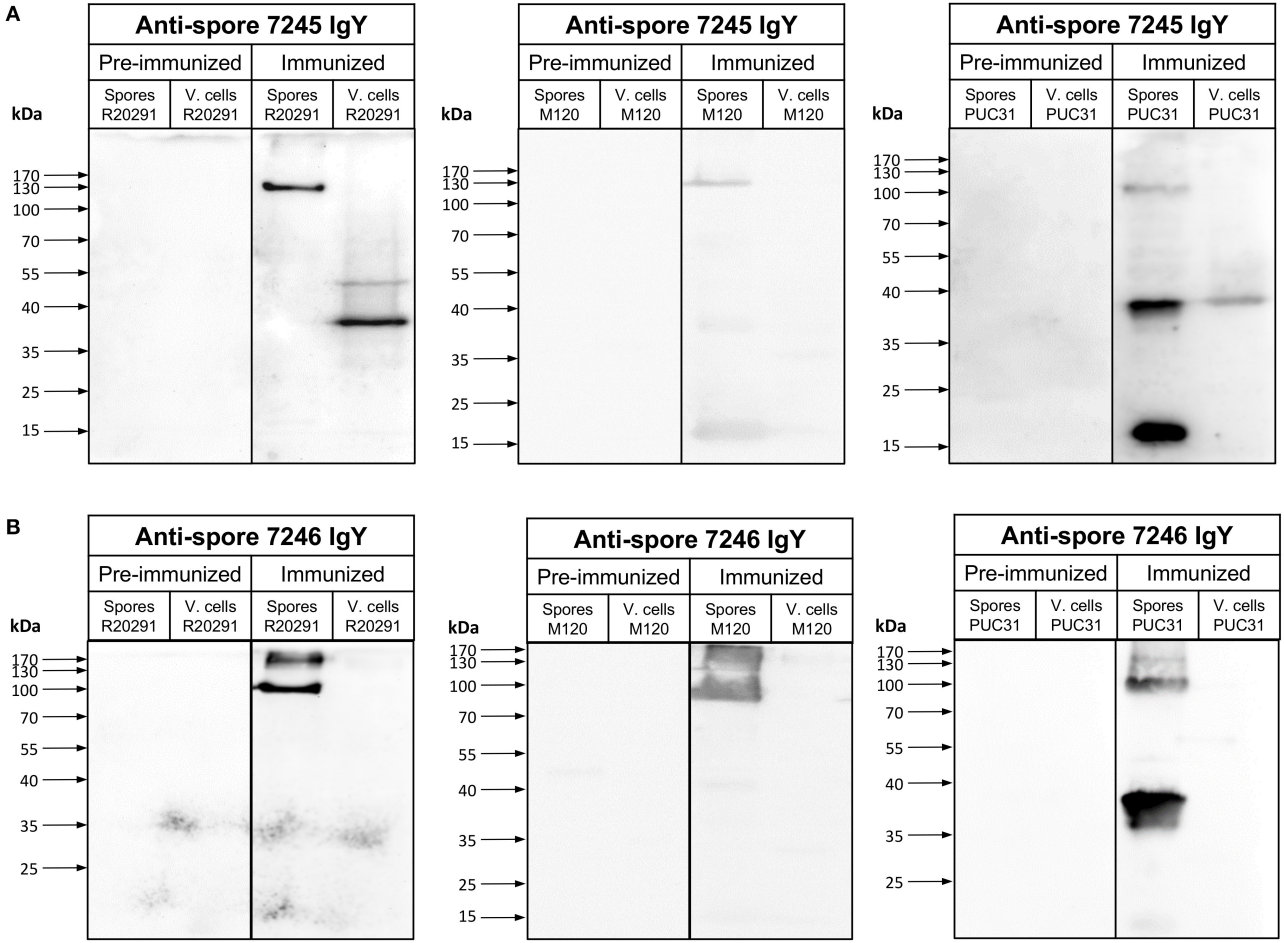
Immunoreactivity of anti-spore IgY against *C. difficile* spores and vegetative cells. **(A,B)** Immunoblotting of spore extracts and vegetative cell lysate (4 × 10^7^ spores or vegetative cells) of various *C. difficile* strains with 1:5000 of preimmunized IgY or anti-spore IgY of batches 7245 **(A)** and 7246 **(B)**. Spore extracts and vegetative cells were separated by 12% SDS-PAGE gel, transferred to nitrocellulose membrane an analyzed by Western blot with IgY prior to immunization (pre-immunized) and anti-spore IgY of batches 7245 **(A)** and 7246 **(B)** as described in the Material and Methods section.

Next, we evaluated the specificity of both batches of anti-spore IgY against *C. difficile* spore extracts and lysates by Western blot analysis. Immunoblotting of R20291 spore extracts with anti-spore IgY of batch 7245 yielded a single immunoreactive band of ~130-kDa (Figure 2A), which contrasts with previously reported immunoreactive species detectable by anti-spore antibodies generated in goat (Barra-Carrasco et al., 2013; Pizarro-Guajardo et al., 2014). Strikingly, upon immunoblotting of R20291 cell lysates of two immunoreactive bands of ~ 50- and 38-kDa were evidenced (Figure 2A), indicating that this batch of anti-spore IgY cross-reacts with *C. difficile* vegetative cells. However, upon evaluation of anti-spore IgY of batch 7246 against spore extracts of R20291 strain, we evidenced that two immunoreactive bands of ~170- and 100-kDa (Figure 2B), which are consistent with previous findings using goat anti-spore IgG (Barra-Carrasco et al., 2013; Pizarro-Guajardo et al., 2014). Immunoblotting of lysates of vegetative cells revealed that anti-spore IgY of batch 7246 does not immunoreact with epitopes of vegetative cells (Figure 2B). By contrast, in immunoblotting R20291 spore extracts and cell lysates with pre-immunized IgY of batches 7245 and 7246 (Figures 2A,B) no immunoreactive bands were detectable, suggesting that immunoreactive protein species detected by the anti-spore IgY are spore-specific antigens.

To expand this analysis, we included spore extracts and vegetative cells of another epidemically relevant PCR ribotype 078 (i.e., M120) which is an epidemic strain first identified in livestock and subsequently identified in clinics across Europe (Goorhuis et al., 2008) and a clinically relevant PCR ribotyope 046 (strain PUC31), isolated from a patient with recurrent infection. Immunoblotting of spore extracts of strain M120 and PUC31 with anti-spore IgY of batch 7245 revealed three immunoreactive bands of ~170-, 37-, and 17-kDa (Figure 2A); however, cross-reactivity with PUC31 cell lysates was evidenced. Upon immunoblotting M120 and PUC31 spores with anti-spore IgY of batch 7246, ~170 and 100-kDa and 100- and 37-kDa, respectively (Figure 2B). This latter batch of IgY did not immunoreact with cell lysates of both strains (Figure 2B). Collectively, these results indicate that: (i) anti-spore IgY of batch 7245 not only detects immunoreactive proteins of spore extracts but also cross-reacts with proteins of cell lysates and, therefore we reasoned that this batch is unsuitable for further experimentation; (ii) anti-spore IgY of batch 7246 uniquely recognizes spore-specific antigens, and therefore was used for further experimentation.

### Characterization of the variability of binding of anti-spore IgY to *C. difficile* spores

**Figure 3:**
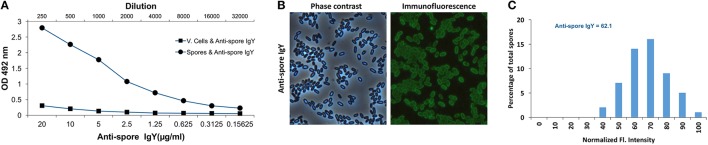
Characterization of immunoreactivity of anti-spore IgY against *C. difficile* spores. **(A)** Immunoreactivity of anti-spore IgY against conformational antigens of *C. difficile* spores and vegetative cells was analyzed by ELISA. **(B,C)** Heterogeneity of binding of anti-spore IgY to *C. difficile* spores. Representative phase contrast and anti-spore immunofluorescence micrographs are shown **(B)**. The fluorescence (Fl.) intensity of 300 spores with anti-spore IgY were analyzed and the distribution of the fluorescence intensity of anti-spore IgY-specific immunofluorescence signal of R20291 spores **(C)**. The median of the fluorescence intensity is shown. The data shown in **(C)** is from one experiment that is representative of three independent experiments.

To further evaluate the specificity of anti-spore IgY of batch 7246, we performed an ELISA to test whether anti-spore IgY cross-reacted against conformational antigens of *C. difficile* vegetative cells. As expected, a significant immunoreactivity of anti-spore IgY was observed against *C. difficile* spores (Figure 3A); by contrast, no significant immunoreactivity was evidenced against vegetative cells, indicating the absence of cross-reactivity of anti-spore IgY with conformational antigens of vegetative cells.

Recent studies demonstrate that *C. difficile* forms spores with two different morphotypes of the outermost exosporium layer in the same sporulating culture: (i) spores with a thin-exosporium layer; and (ii) spores with a thick exosporium layer (Pizarro-Guajardo et al., 2016a,b). These studies have demonstrated that ~75% of the spores have a thin exosporium layer, while 25% of the spores have a thick exosporium layer. Therefore, we also assessed by indirect immunofluorescence the heterogeneity of binding of anti-spore IgY to heterogeneous spore population. Analysis of 300 spores revealed that 100% of the spores yielded immunofluorescence signal specific for anti-spore IgY (Figure 3B). Upon quantitative analysis of the immunofluorescence signal revealed an average relative fluorescence intensity of 64 arbitrary units for spores incubated with anti-spore IgY (Figure 3C). Analysis of the distribution of the fluorescence intensity evidenced that anti-spore IgY yielded a near normal distribution of the fluorescence intensity (Figure 3C). Collectively, these observations suggest that, despite the ultrastructural differences reported in R20291 spores, anti-spore IgY binds to both morphotypes with a normal distribution.

### Opsonization of *C. difficile* spores with anti-spore IgY does not affect germination

**Figure 4:**
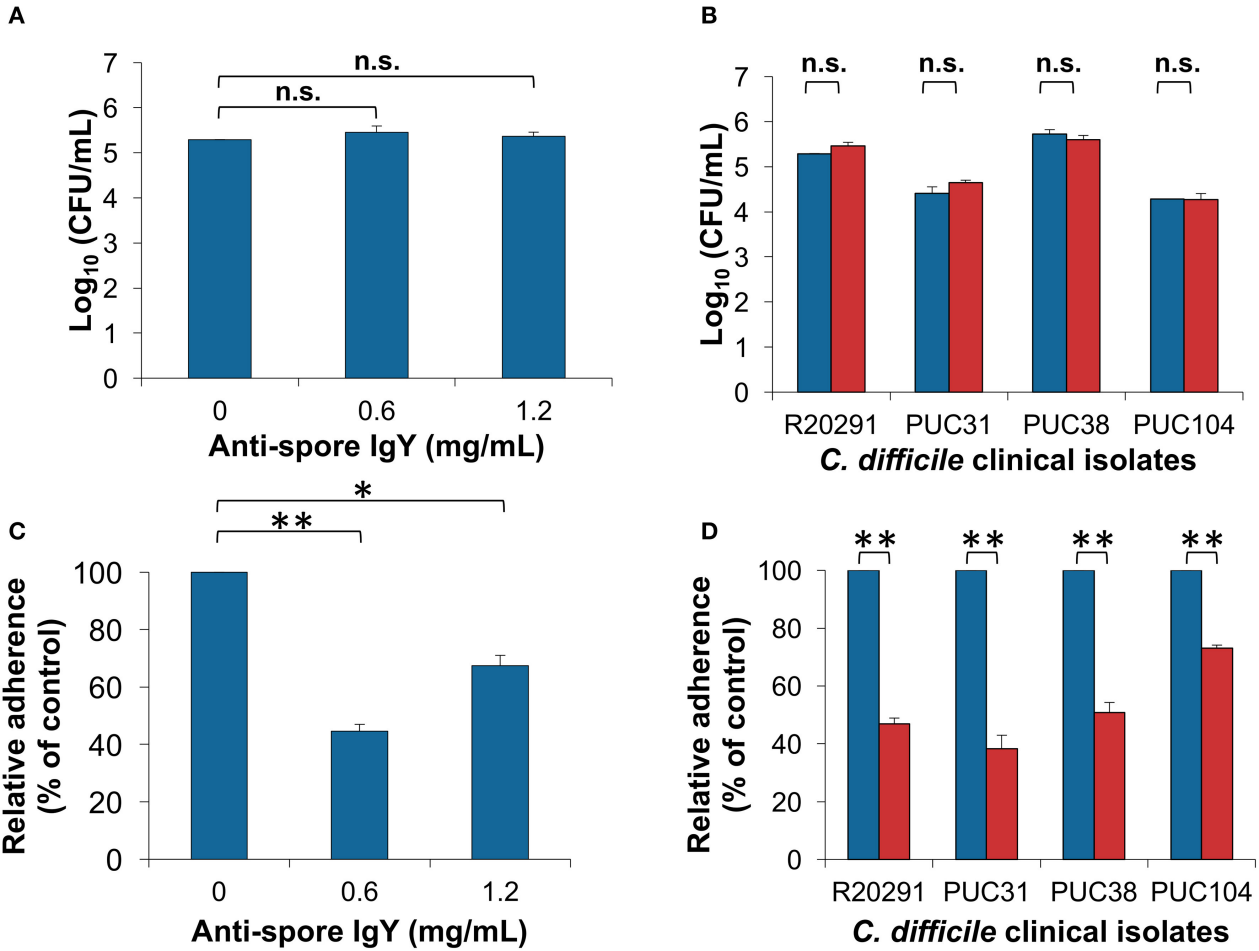
Effect of anti-spore IgY on *C. difficile* spore-colony formation and adherence to intestinal epithelial cells. **(A)** Effect of various concentrations of anti-spore IgY on the efficiency of *C. difficile* R20291 spores to form colonies as a measure of germination, colony forming units were determined in BHIS agar plates supplemented with taurocholate (ANOVA test followed by Tukey's posttest). **(B)** Effect of anti-spore IgY on the germination efficiency of several *C. difficile* clinical isolates. Untreated (blue bars) and anti-spore IgY (0.6 mg/mL)-treated spores (red bars) were plated on BHIS agar plates supplemented with taurocholate and colony forming units counted (Paired *t*-test, n.s., no significance). **(C)** Effect of various concentrations of anti-spore IgY on spore-adherence of *C. difficile* R20291 spores to differentiated Caco-2 cells (Student *t*-test, ^*^P = 0.001; ^**^P = 0.0001). **(D)** Effect of anti-spore IgY on the adherence of spores of various clinical isolates of *C. difficile* to differentiated Caco-2 cells (Paired *t*-test, n.s, *P* > 0.05). Blue bars, untreated spores; red bars, anti-spore IgY-treated spores. Data represents the mean of three independent experiments and error bars are standard error of the mean.

Next, we evaluated whether opsonization of *C. difficile* spores would affect their germination ability when incubated at saturated concentrations of IgY. *C. difficile* R20291 spores incubated with anti-spore IgY at concentrations of saturation caused no significant decrease in the efficiency of spore-colony formation when plated on BHIS agar plates in presence of sodium taurocholate (BHIS+ST) (Figure 4A) (ANOVA test followed by Dunn's posttest). The effectiveness in inhibition of germination was also tested in clinical *C. difficile* isolates; spores from Chilean isolates PUC31, PUC38, and PUC104 (Plaza-Garrido et al., 2015) were pre-incubated with anti-spore IgY and plated in BHIS+ST. No significant decrease in the spore colony forming ability was observed in IgY-opsonized spores of clinical Chilean strains with anti-spore IgY (Figure 4B). From these results, we conclude that opsonization of *C. difficile* spores with anti-spore IgY does not affect spore viability.

### IgY-opsonized *C. difficile* spores exhibit reduced adherence to intestinal epithelial cells

We evaluated if opsonization of *C. difficile* spores with anti-spore IgY affects spore adherence to epithelial cells. *C. difficile* R20291 spores incubated with saturated and twice saturated concentration of IgY, presents a significant decrease in spore-adherence to differentiated Caco-2 cells, estimated in ~44% when opsonized with saturation concentration of anti-spore IgY (Figure 4C). Notably, 2-fold increase in the saturation concentration of anti-spore IgY lead to an increase in spore adherence to 68% (Figure 4C), suggesting the formation of immune complexes that rather than inhibiting, might be favoring spore adherence. These results, with the optimum concentration of anti-spore IgY, were further expanded by inclusion of three *C. difficile* clinical isolates (i.e., PUC31, PUC38, and PUC104). Opsonization of spores of these three clinical isolates also evidenced reduced spore-adherence to intestinal epithelial cells to a final extent of ~38 and 73% of total spores when incubated with saturated amounts of IgY anti-spore, depending on the isolate (Figure 4D). Collectively, these results indicate that anti-spore IgY can reduce the adherence of various clinical isolates to intestinal epithelial cells.

### Analysis of the immunoreactivity of anti-spore IgY against denatured and conformational antigens of the murine microbiota

**Figure 5:**
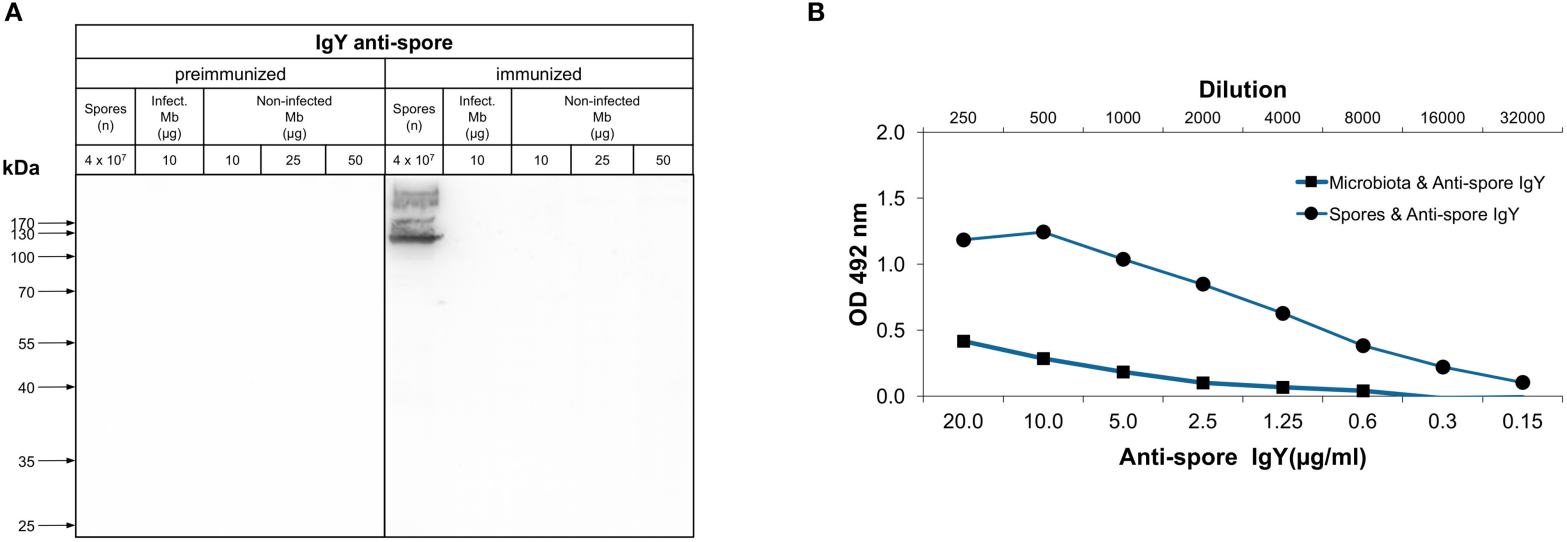
Immunoreactivity of anti-spore IgY against murine microbiota. **(A)** Western blot analysis of immunoreactivity of IgY against microbiota lysates. Bacterial cells from fecal microbiota (Mb), *C. difficile* infected-mice (Positive micro), and microbiota mixed with R20291 spores (10^8^/ml) were electrophoresed, transferred to a nitrocellulose membrane and probed with pre-immunized and anti-spore IgY. *C. difficile* R20291 spores were used as a positive control. No immunoreactive band was observed in Positive micro, presumably due to low relative abundance of *C. difficile* spores in bacterial cell lysates. **(B)** Immunoreactivity of anti-spore IgY against conformational antigens of bacterial cells of the fecal microbiota of mice was detected by ELISA. As a positive control, *C. difficile* R20291 spores were used.

An important feature of an orally administered immunotherapy is that the antibodies should not bind to bacterial cells of the host microbiota, minimizing it's impact in the gut microbiome. Consequently, we evaluated whether the anti-spore IgY of batch 7246 immunoreacted with denaturized antigens of bacterial cells derived from mice microbiota of healthy mice (non-infected Mb) and *C. difficile* infected-mice (infect. Mb). Western blot analysis with pre-immunized IgY revealed absence of immunoreactive protein species in extracts of *C. difficile* spores and bacterial cells of microbiota from *C. difficile* infected and non-infected mice (Figure 5A). Western blot analysis with anti-spore IgY show no immunoreactivity against proteins present in both bacterial cell extracts (Figure 5A). To further confirm the absence of cross-reactivity of anti-spore IgY against murine microbiota's bacterial cells, immunoreactivity against conformational antigens was assessed by ELISA. As expected, a high affinity of anti-spore IgY was observed against *C. difficile* spore extracts, while a lower affinity of anti-spore IgY was evidenced against bacterial cells of the murine microbiota (Figure 5B). Based on these results we speculated that oral administration of anti-spore IgY should be specific against *C. difficile* spores with a minimal impact against murine gut microbiota.

### Oral administration of 600 μg of anti-spore IgY prior to *C. difficile* spore infection prevents disease initiation and reduced *C. difficile* spores in colonic tissue

Based on previous studies that administered IgY doses range from 50 to 100 μg (Liou et al., 2010; Nguyen et al., 2010), we first evaluated the effect of 100 and 200 μg of IgY per dose as prophylactic treatment. However, no difference in body weight, time to diarrhea development, score of diarrhea, spore shed through feces, cecum toxin titer was evidenced between untreated and IgY-treated mice (Figure S2). Depending on the infectious dose, the infection with *C. difficile* R20291 spores has been described to be associated with low mortality (Winston et al., 2016) and also to cause mild clinical manifestations that lead to self-recovery of the animals (Zackular et al., 2016). Differences between studies may also be explained by the fact that gut microbiota has a high inter-laboratory variation as has been recently evidenced (Lagkouvardos et al., 2016). In this work, we observed that all of the mice develop mild-diarrhea and we did not observe moribund animals before the endpoint of the experiments.

**Figure 6:**
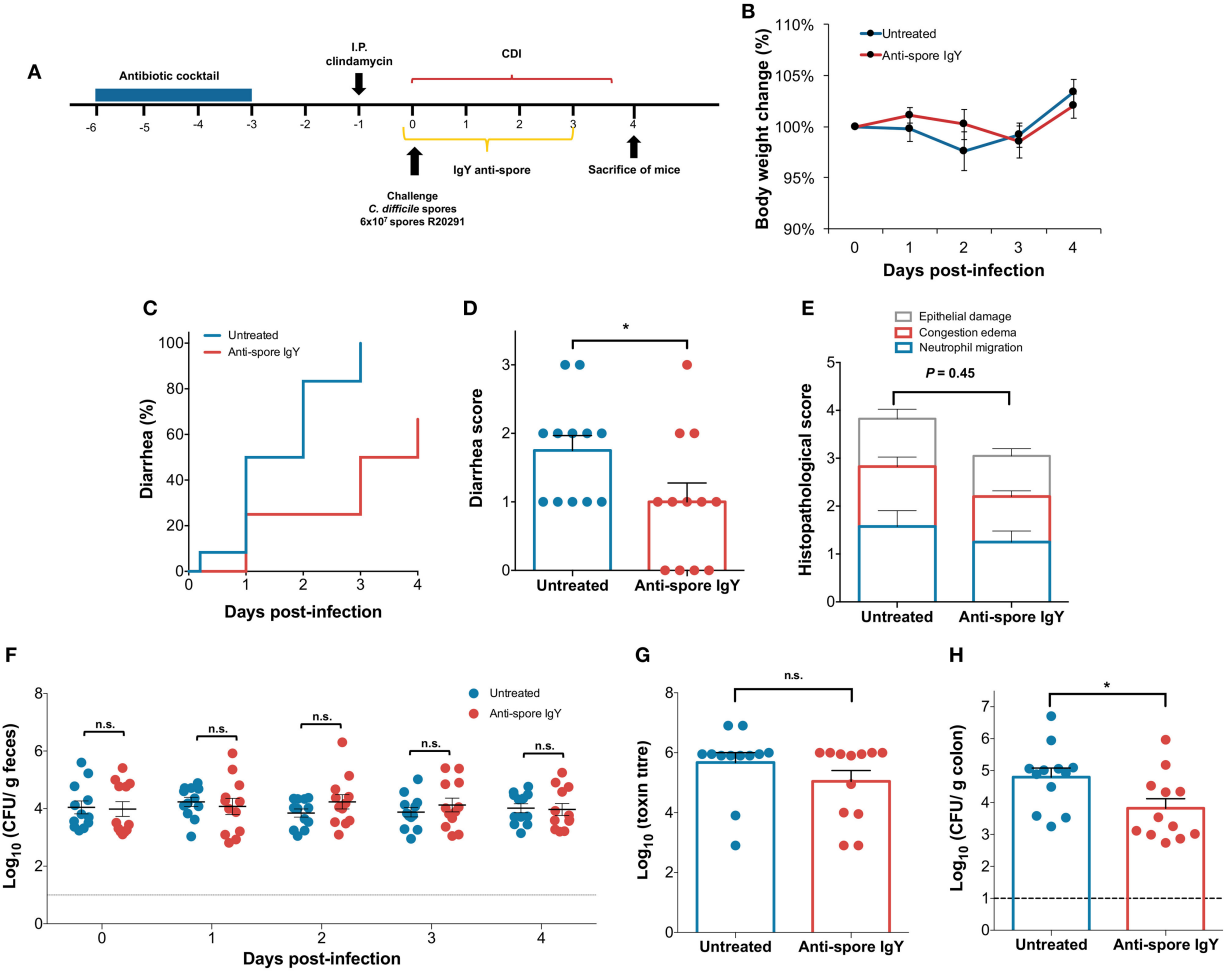
Administration of 600 μ g of anti-spore IgY during the initiation of CDI. **(A)** Overview of the experimental design schematics for the prevention of initiation of *C. difficile* infection in a murine model. Antibiotic treated C57BL/6 mice were infected with *C. difficile* R20291 spores (6 × 10^7^ spores; *n* = 12 per group) and subsequently treated with oral administration of anti-spore IgY or phosphate buffer saline as a control for 3 days. **(B)** Body weight change was slightly higher in untreated mice (blue line) and mice treated with anti-spore IgY (red line). Untreated and anti-spore IgY-treated *C. difficile*-challenged mice were monitored for: **(C)** time to diarrhea; **(D)** score of diarrhea; **(E)** Colonic histological scores; **(F)** Fecal *C. difficile* spore shedding; **(G)** Cecum content cytotoxicity; **(H)** *C. difficile* spores in colonic tissue. Data of Figure 7 is representative of two independent experiments. Error bars are standard error of the mean. n.s., is no significance; ^*^P ≤ 0.05.

**Figure 7:**
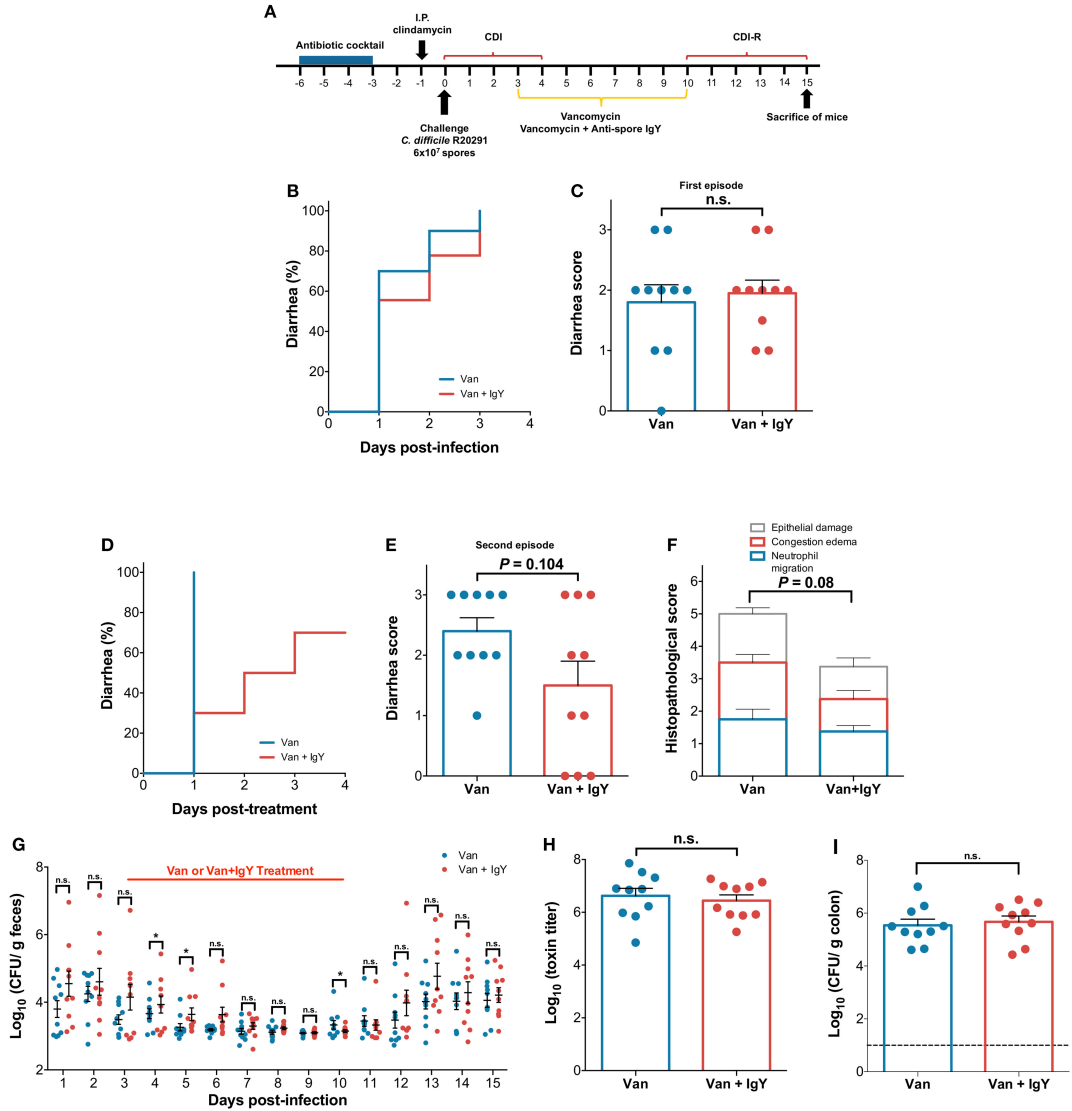
Oral administration of anti-spore IgY in combination with vancomycin delays the occurrence of recurrent CDI in a murine model. **(A)** Overview of the experimental design schematics for the prevention of the recurrence of *C. difficile* infection in a murine model. Antibiotic treated C57BL/6 mice were infected with *C. difficile* R20291 spores (6 × 10^7^ spores; *n* = 10 per group) and subsequently orally treated with anti-spore IgY and vancomycin or with vancomycin alone for 7 days. *C. difficile*-challenged mice treated with vancomycin or Vancomycin/IgY were monitored for: **(B)** time to diarrhea during first episode; **(C)** score of diarrhea during first episode; **(D)** time to diarrhea during recurrence of CDI; **(E)** diarrhea score during recurrence of CDI; **(F)** Colonic histological scores during recurrence of CDI; **(G)** Fecal *C. difficile* spore shedding; **(H)** Cecum content cytotoxicity; **(I)** *C. difficile* spores in colonic tissue. Data of Figure 7 is representative of two independent experiments. Error bars are standard error of the mean. n.s., is no significance; ^*^P ≤ 0.05.

Since administration of 200 μg of anti-spore IgY did not delay the initiation of CDI, we evaluated whether a 3-fold increase in the dose of orally administered anti-spore IgY could delay the initiation of CDI. Antibiotic-treated C57BL/6 mice were challenged with 6 × 10^7^ spores (*n* = 12 per treatment), clinical symptoms were monitored daily, and fecal samples, cecum content and colonic tissue were collected (Figure 6A). A slight but not significant difference in body weight loss was observed between untreated- and anti-spore IgY-treated mice during the first 3 days after infection (Figure 6B). Notably, administration of anti-spore IgY caused a significant delay of 2 days in the appearance of diarrheic symptoms (Log-rank test, *P* ≤ 0.01) (Figure 6C); the median time to diarrhea was 1.5 days for untreated mice and 3.5 days for anti-spore IgY-treated mice (Figure 7C). The score of diarrhea was also lower (Student *t*-test, *P* = 0.0429) in anti-spore IgY-treated mice than in untreated mice (Figure 6D). Despite a slight decrease in the overall histopathological score (Mann-Whitney unpaired, *P* = 0.45), no significant difference was observed between untreated (3.83 ± 0.7) and anti-spore IgY-treated mice (3.0 ± 0.4) (Figure 6E and Figure S3). No significant difference was evidenced in the levels of spores shedded in feces between untreated and anti-spore IgY-treated mice at 0, 1, 2, 3, and 4-day post-infection (Mann-Whitney unpaired test, day 0 *P* = 0.86; day 1 *P* = 0.062; day 2 *P* = 0.19; day 3 *P* = 0.41; day 4 *P* = 0.86) (Figure 6F). Administration of anti-spore IgY had no effect on cytotoxic titers of cecum contents, with no significant difference (*P* = 0.65) observed between untreated and anti-spore IgY treated mice (Figure 6G). Strikingly, administration of anti-spore IgY caused a significant reduction (*P* = 0.03) of ~90% of *C. difficile* R20291 spores in colonic tissue vs. untreated mice (Figure 6H). Collectively, these observations indicate that administration of anti-spore IgY prior and during infection, delays the initiation of CDI and reduces the spore-load in colonic tissue.

### Effect of oral co-administration of 600 μg of anti-spore IgY with vancomycin during a recurrence model of CDI

Given the ability of anti-spore IgY to reduce adhered spores to colonic tissue, we sought to evaluate whether administration of anti-spore IgY in combination with vancomycin would delay the development of recurrence. Consequently, we used a mouse model of recurrence, where infection recurrence can be induced after a 7-day regime of orally administered vancomycin 3 days post-infection. A group of *C. difficile*-challenged mice (*n* = 12) were treated with vancomycin (control for recurrence) and another group of *C. difficile*-challenged mice (*n* = 12) were treated with vancomycin and anti-spore IgY (Vancomycin/IgY) (Figure 7A). No difference in the time to the first episode of diarrhea (Log-rank, *P* = 0.45) (Figure 7B) and overall score of diarrhea (Mann-Whitney unpaired, *P* = 0.88) (Figure 7C) were observed post-*C. difficile*-challenge between both groups of mice. However, after administration of a 7-day regime of vancomycin, 100% of vancomycin treated mice exhibited diarrhea after 1 day-post vancomycin treatment (Figure 7D). By contrast, only 30% of Vancomycin/IgY-treated mice exhibited diarrhea after 1-day post Vancomycin/IgY-treatment (Figure 7D). The co-administration of Vancomycin/IgY caused a delay in the occurrence of diarrhea by a median of 1.5 days (*P* = 0.0021) (Mann Whitney unpaired *t*-test; *P* = 0.104) (Figure 7D). Histological score of mice treated with Vancomycin/IgY (3.3 ± 0.6) was slightly lower (Unpaired *t*-test with Welch's correction; *P* = 0.08) compared to mice treated with vancomycin alone (5.0 ± 0.6) (Figure 7F and Figure S3), which could be attributed to the lower tissue damage and prevention of the recurrence of the disease. Based on these observations, we suggest that co-administration of Vancomycin/IgY reduces the recurrence of diarrhea.

*C. difficile* spore-load in feces, cecum content cytotoxicity and spores adhered to colonic tissue were also evaluated. Wilcoxon matched-pairs signed rank test revealed significant differences (*P* = 0.002) in *C. difficile* spores shed in feces between vancomycin- and Vancomycin/IgY-treated mice (Figure 7G). A significant increase in *C. difficile* spores in feces was observed at day 4 and 5 after infection; at day 4, mice treated with vancomycin and Vancomycin/IgY released 3.63 and 3.93, respectively (Mann-Whitney unpaired test, *P* ≤ 0.05), while at day 5, fecal shedding for vancomycin and Vancomycin/IgY-treated mice was of 3.21 and 3.63 Log CFU, respectively (Mann-Whitney unpaired test, *P* ≤ 0.05). Notably, at the end of treatment (i.e., day 10), vancomycin and Vancomycin/IgY-treated mice had fecal shedding levels of 3.22 and 3.14 Log CFU, respectively (Mann-Whitney unpaired test, *P* ≤ 0.05), suggesting that administration of anti-spore IgY contributed to the increased fecal spore-shedding, presumably due to a dual effect of vancomycin targeting vegetative cells and anti-spore IgY targeting *C. difficile* spores. No significant difference in cecum content cytotoxicity between vancomycin (6.6 ± 0.3) and Vancomycin/IgY-treated (6.4 ± 0.2) mice 5 days after treatment was evidenced (Mann-Whitney unpaired test, *P* = 0.62) (Figure 7H). Similarly, no significant difference in the number of spores adhered to colonic tissue (Mann-Whitney unpaired test, *P* = 0.69) between both groups of mice treated with vancomycin (5.5 ± 0.23) and Vancomycin/IgY (5.7 ± 0.22) was evidenced (Figure 7I). Collectively, these results suggest that rapid outgrowth of *C. difficile* occurs after Vancomycin/IgY treatment, leading to recolonization and toxin production in the cecum.

### No IGG response to IgY and *C. difficile* spores was observed in the mice during the course of CDI in initiation and recurrence model

We also analyzed whether IgG against the administered IgY as treatment were raised. Serum from *C. difficile*-challenged mice treated with anti-spore IgY (initiation group) and Vancomycin/IgY (recurrence group) and untreated mice (naive). No immunoreactivity against IgY was evidenced between all three groups of mice (Kruskal-Wallis ANOVA, *P* = 0.602) (Figure S4A). No significant difference in serum reactivity against *C. difficile* R20291 spores between these groups of mice (i.e., naive, Initiation and recurrence) (Kruskal-Wallis ANOVA, *P* = 0.082) (Figure S4B) was observed. These results demonstrate that during the treatment to prevent initiation and recurrence models of the infection, no significant immune response was generated against IgY administered as treatment or *C. difficile* spores during the treatment of the initiation and recurrence models of the infection.

## Discussion

Passive immunization is the transfer of active humoral immunity in the form of ready-made antibodies. Some mechanisms of host protection by oral administration of IgY have been proposed and include: (i) inhibition of pathogen adhesion to cell surfaces; (ii) bacterial agglutination and flushed down the gut (Rahman et al., 2013). Therefore, given that *C. difficile* spores play an important role in the initiation and persistence of the infection, the goal of this study was to evaluate: (i) whether IgY could be specifically raised against *C. difficile* spores; and (ii) oral administration of anti-spore IgY during initiation and recurrence of CDI. In this context, our results provide several noteworthy observations that are further discussed.

This work demonstrates that significant differences in *C. difficile* spore-specific IgY obtained from two hens. Titers of batch 7245 were 2-fold lower than batch 7246, which could be attributed to the differences in the immunoreactive species detected by each batch (Figure 2). Although the reasons for these differences are unclear, the fact that IgY of batch 7245, but not it's pre-immunized IgY, immunoreacted with vegetative cells suggests that during immunization, this chicken was exposed to *C. difficile* vegetative cells. Due to these uncertainties, IgY from batch 7245 was discarded from further experiments. It was also noteworthy that despite the elevated number of protein species present in the spore surface (i.e., ~180) (Díaz-González et al., 2015), only two species of molecular masses of ~170- and 100-kDa were found to be immunoreactive against anti-spore IgY (batch 7246) (Figure 2). Although the nature of these immunoreactive protein species is unclear, their molecular masses seems to be strain dependent, suggesting differences between strains in the assembly of the outermost layers. These proteins also seem to be abundant in the spore surface of strain R20291, as evidenced by the elevated number of IgY molecules that saturate the spore surface (~70,000 molecules per spore). Further work oriented to identify these immunoreactive proteins is underway.

A notable observation of this work is that anti-spore IgY binds to the entire spore population. Recently, several studies have highlighted that during sporulation, *C. difficile* forms spores with two different exosporium morphotypes: (i) spores with an exosporium layer which is thin in the amount of electron-dense material that surrounds the spore coat; and (ii) spores with an exosporium layer thick in electron-dense material surrounding the spore coat (Pizarro-Guajardo et al., 2016a,b). The relative abundance of each exosporium morphotype is strain-dependent; in the case of strain R20291, spores with a thin- and thick-exosporium morphotype represent ~75 and 25% of the spore population, respectively (Pizarro-Guajardo et al., 2016a,b). In R20291 spores, both types of spore have hair-like extensions, which is a typical feature of epidemically relevant strains (Pizarro-Guajardo et al., 2016a). In this context, and despite these ultrastructural differences, quantitative analysis of the immunofluorescence intensity reveals a normal distribution, indicating that the immunoreactive protein specie or species are present in the entire spore population and therefore, their presence is likely not affected by the ultrastructural variability observed in the exosporium layer of *C. difficile* spores.

A main conclusion of this work is that anti-spore IgY reduces spore adherence to intestinal epithelial cells. The mechanism(s) of adherence of *C. difficile* spores to intestinal epithelial cells remains elusive; several reports suggest that spore ligand(s) and cellular receptor(s) are involved but have failed to identify such molecules (Paredes-Sabja and Sarker, 2012; Mora-Uribe et al., 2016). Notably, despite the saturation of *C. difficile* spore surface with an elevated number of anti-spore IgY molecules, spore-adherence to intestinal cells only decreased by ~50%, suggesting that opsonization of the spore surface with anti-spore IgY does not completely block spore-ligand(s) involved in spore-adherence to intestinal cells. This suggests that anti-spore IgY immunoreacts with spore-surface epitopes that are not involved in spore adherence and that are present in various clinically relevant *C. difficile* strains. A previous report suggested that the hair-like extensions of the spore surface might be involved in spore-adherence (Mora-Uribe et al., 2016); therefore, IgY antibodies against these structures might inhibit spore-adherence more efficiently. Further experimentation aiming to determine whether these hair-like extensions are made of the collagen-like BclA2 and/or BclA3 by using transmission electron microscopy and immunogold antibodies will provide conclusive evidence of their composition and aid in the development of IgY-based therapies.

The approach of using specific IgY for prevention and treatment of CDI offers an alternative to current immunotherapies, which use intravenous administration of anti-TcdA and TcdB monoclonal human antibodies (Negm et al., 2017). To the best of our knowledge, there are no reported prophylactic therapies that target to prevent the initiation of CDI in a susceptible host by removing *C. difficile* spores from the host. In this context, here, we provide evidence that a spore-oriented therapy using chicken IgY, from *C. difficile* spore-immunized hens, administered in a prophylactic manner prior and during the infection can delay the initiation of CDI. The effect of oral administration of 600 μg per dose of anti-spore IgY as prophylactic therapy contributed mainly to delay the initiation of diarrhea and to reduce the spore load in colonic tissue. Initial doses of 100 and 200 μg of IgY, administered in 50 μl at 2 and 4 mg/ml which were ~3 and 6-fold higher than the *in vitro* saturation concentration, were ineffective. This could be attributed to: (i) *in vivo* dilution of IgY below an effective concentration to efficiently opsonize the spore; and/or (ii) to proteolytic degradation of IgY by digestive enzymes. Despite the drawbacks in the administration format used in this work, we observed that increasing the dose to 600 μg (i.e., 12 mg/ml) was sufficient to cause a delay in the initiation of diarrhea. It was notable to observe that administration of IgY had no impact in fecal shedding of *C. difficile* spores, which could be attributed by anti-spore IgY being unable to recognize sporulating cells. A major drawback of oral administration of anti-spore IgY was the negligible decrease in cecum content cytotoxicity. The data also demonstrates that administration of anti-spore IgY reduces the spore load in the colonic tissue; however, this might not be the case for *C. difficile* spores in cecum contents, were no reduction in cytotoxicity was detectable during IgY treatment. The latter suggests that opsonized spores able to germinate and release the nascent cell are capable of colonizing the cecum and establishing the infection. Our *in vitro* results demonstrate that opsonization of *C. difficile* spores with anti-spore IgY has no effect on spore colony forming efficiency and support this notion. In the GI tract of antibiotic-treated mice, *C. difficile* spores encounter favorable conditions of bile acids that trigger spore germination and outgrowth (Buffie et al., 2015). To overcome this limitation, oral administration of anti-spore IgY could be combined with taurocholate analogs shown to inhibit spore germination and progression of the disease in mouse models (Sorg and Sonenshein, 2009; Howerton et al., 2013). In summary, further optimization of IgY based oral immunotherapy could include a mixture of IgY specific against spores, vegetative cells and toxins, and bile acid analogs inhibitors of spore germination.

A major clinical challenge in CDI treatment is the elevated rate of recurrence of the infection (Evans and Safdar, 2015). *C. difficile* sporulation during infection is essential for the recurrence of the disease (Deakin et al., 2012); current antibiotic-therapies (i.e., metronidazole and vancomycin) to treat *C. difficile* infections have effect on the spores being produced during the infection which can persist and cause infection recurrence. In this context, we demonstrate that administration of anti-spore IgY in combination with vancomycin during CDI treatment delays the appearance of diarrhea. Notably the impact of this combined therapy on the histopathological score was modest presumably because most of the histological damage occurred during the first episode of the infection and also due to the lack of differences in cecum content cytotoxicity. The timing of the measurements (day 5 after IgY-vancomycin treatment) could have affected our results, allowing *C. difficile* to re-colonize, release toxins and sporulate leading to negligible differences in the latter phenotypes. Unfortunately, in this work we did not evaluate the dynamics of removal of *C. difficile* spores from colonic tissue during IgY-vancomycin nor the composition/structure of the microbiota, which could have provided a deeper insight on the effects of oral administration of specific IgY.

In summary, in this work we demonstrate that highly specific chicken IgY antibodies can be raised against *C. difficile* spores, with minimal cross-reactivity with bacterial cells of the murine microbiota. Notably, we provide experimental evidence that suggest that the administration of anti-spore IgY can be used for two different purposes (i) as a prophylactic therapy when administered orally in susceptible patients prior to the initiation of the infection, to delay the initiation of *C. difficile*-associated diarrhea; (ii) in combination with vancomycin, to delay the appearance of recurrent diarrhea. Although the IgY based treatments shown in this work can be clearly optimized, we believe that this study provides a starting platform for further development of alternative passive immunotherapies to combat CDI and help reduce the incidence of CDI and of recurrent CDI.

## Ethics statement

This study was carried out in accordance with the recommendations of Bioethical guidelines of CONICYT. The protocol was approved by the Comité; de Bioética de la Universidad Andrés Bello.

## Author contributions

MP, FD, MA, and DP designed the experiments. MP and FD performed the experiments. MP, FD, MA, and DP analyzed and interpreted data. MP, MA, and DP wrote the manuscript with input of co-authors. MP, FD, MA, and DP critically revised the manuscript.

### Conflict of interest statement

The authors declare that the research was conducted in the absence of any commercial or financial relationships that could be construed as a potential conflict of interest.

## Supplementary material

Figure S1 Presence of an alternative splicing variant of the heavy chain and scheme of the purification protocol of full-length IgY through salting out under acidic conditions. **(A,B)** SDS-PAGE **(A)** and western blot **(B)** analysis of the IgY suspensions obtained from AvesLab. Total proteins of egg yolk (control) and AvesLab IgY suspension of batches 7245 and 7246 were electrophoresed and stained with Coomassie G250. Arrows indicate the position of the full length heavy chain (Fl-HC), the alternative splicing variant of the heavy chain (Δ-HC), and light chain of IgY (LC) of molecular mass of ~75-80-, 38-, and 23-kDa, respectively. **(C)** Purification protocol of fill-length IgY. IgY suspension (1) is diluted 1:20 with saline solution (2), followed by pH adjustment to 4.0 (3) and centrifugation at 3,700 g for 20 min at 4°C (4). Next, the pellet, containing precipitated full-length IgY, is resuspended in 1 ml of PBS (5) filter-sterilized, and aliquots stored at −20°C until use (adapted from: (Hodek et al., 2013)).

Figure S2 Administration of 100 and 200 μg of anti-spore IgY during the initiation of CDI. **(A)** Overview of the experimental design schematics for the prevention of initiation of *C. difficile* infection in a murine model. Antibiotic treated C57BL6 mice were infected with *C. difficile* R20291 spores (6 × 10^7^ spores; *n* = 3 per group) and subsequently treated with oral administration of anti-spore IgY or phosphate buffer saline as a control for 3 days. **(B)** Body weight change of untreated mice (blue line) and mice treated with 100 μg (red line) and 200 μg (green line) of anti-spore IgY and with 200 μg of pre-immunized IgY (gray line). *C. difficile*-challenged mice were monitored for: **(C)** time to diarrhea; **(D)** score of diarrhea; **(E)** Fecal *C. difficile* spore shedding; **(F)** Cecum content cytotoxicity at sacrifice; Data are representative of two independent experiments. Error bars are standard error of the mean. n.s., no significance; ^*^*P* ≤ 0.05; ^**^*P* ≤ 0.01. **(D,F)** Un, untreated; 100, is 100 μg of anti-spore IgY; 200, is 200 μg of anti-spore; Pre 200, is 200 μg of pre-immunized anti-spore IgY.

Figure S3 Histological examination of colonic tissues obtained from antibiotic treated mice exposed to *C. difficile*. Micrographs show colonic tissue of: **(A)** Non-infected mouse colon; **(B)** Colon from mice infected with *C. difficile* (control in initiation model); **(C)** Colon from mouse treated with IgY anti-spore prior and after infection (initiation model); **(D)** Colon from mouse infected and treated with vancomycin (recurrence model); **(E)** Colon from mouse infected and treated with vancomycin combined with IgY anti-spore. Black arrow shows edema, and arrow head shows neutrophilic recruitment.

Figure S4 Serum titers raised against IgY and *C. difficile* spores during initiation and recurrence of CDI. Serum of naive mice, as a control, mice of the infection experiment (infection), and mice of the recurrent infection experiment (recurrence) were isolated and reactivity against *C. difficile* spores **(A)** and anti-spore IgY **(B)** was analyzed by ELISA as described in Methods.
