# Supplementary figures and images for: Characterization of Chicken IgY Specific to Clostridium difficile R20291 Spores and the Effect of Oral Administration in Mouse Models of Initiation and Recurrent Disease
Source: Front Cell Infect Microbiol. 2017 Aug 14;7:365. doi: 10.3389/fcimb.2017.00365 (PMC5557795; doi:10.3389/fcimb.2017.00365)

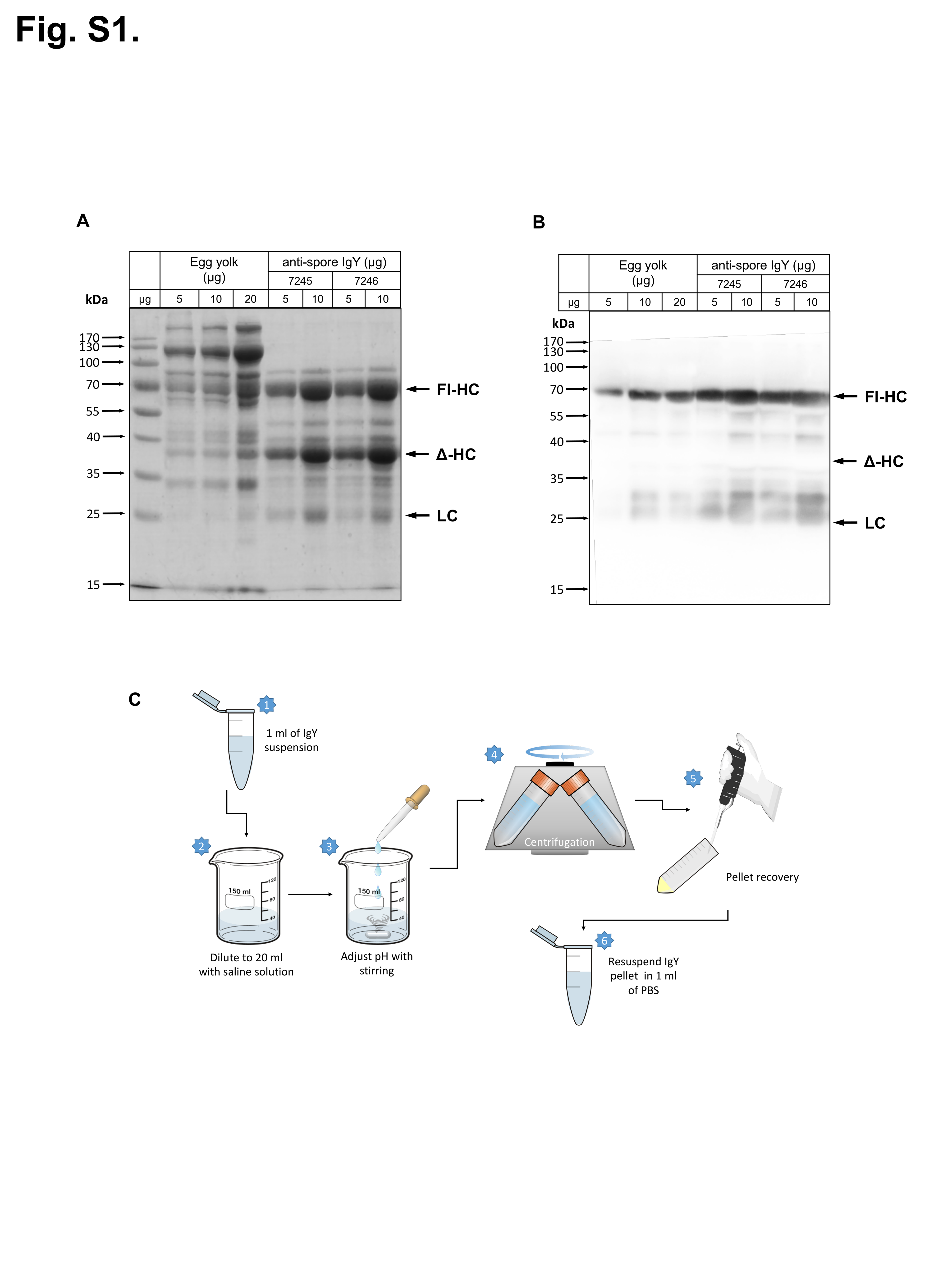

Supplement: Figure S1 — Presence of an alternative splicing variant of the heavy chain and scheme of the purification protocol of full-length IgY through salting out under acidic conditions. (A,B) SDS-PAGE (A) and western blot (B) analysis of the IgY suspensions obtained from AvesLab. Total proteins of egg yolk (control) and AvesLab IgY suspension of batches 7245 and 7246 were electrophoresed and stained with Coomassie G250. Arrows indicate the position of the full length heavy chain (Fl-HC), the alternative splicing variant of the heavy chain (Δ-HC), and light chain of IgY (LC) of molecular mass of ~75-80-, 38-, and 23-kDa, respectively. (C) Purification protocol of fill-length IgY. IgY suspension (1) is diluted 1:20 with saline solution (2), followed by pH adjustment to 4.0 (3) and centrifugation at 3,700 g for 20 min at 4°C (4). Next, the pellet, containing precipitated full-length IgY, is resuspended in 1 ml of PBS (5) filter-sterilized, and aliquots stored at −20°C until use (adapted from: (Hodek et al., 2013)). [file Image1.TIFF]

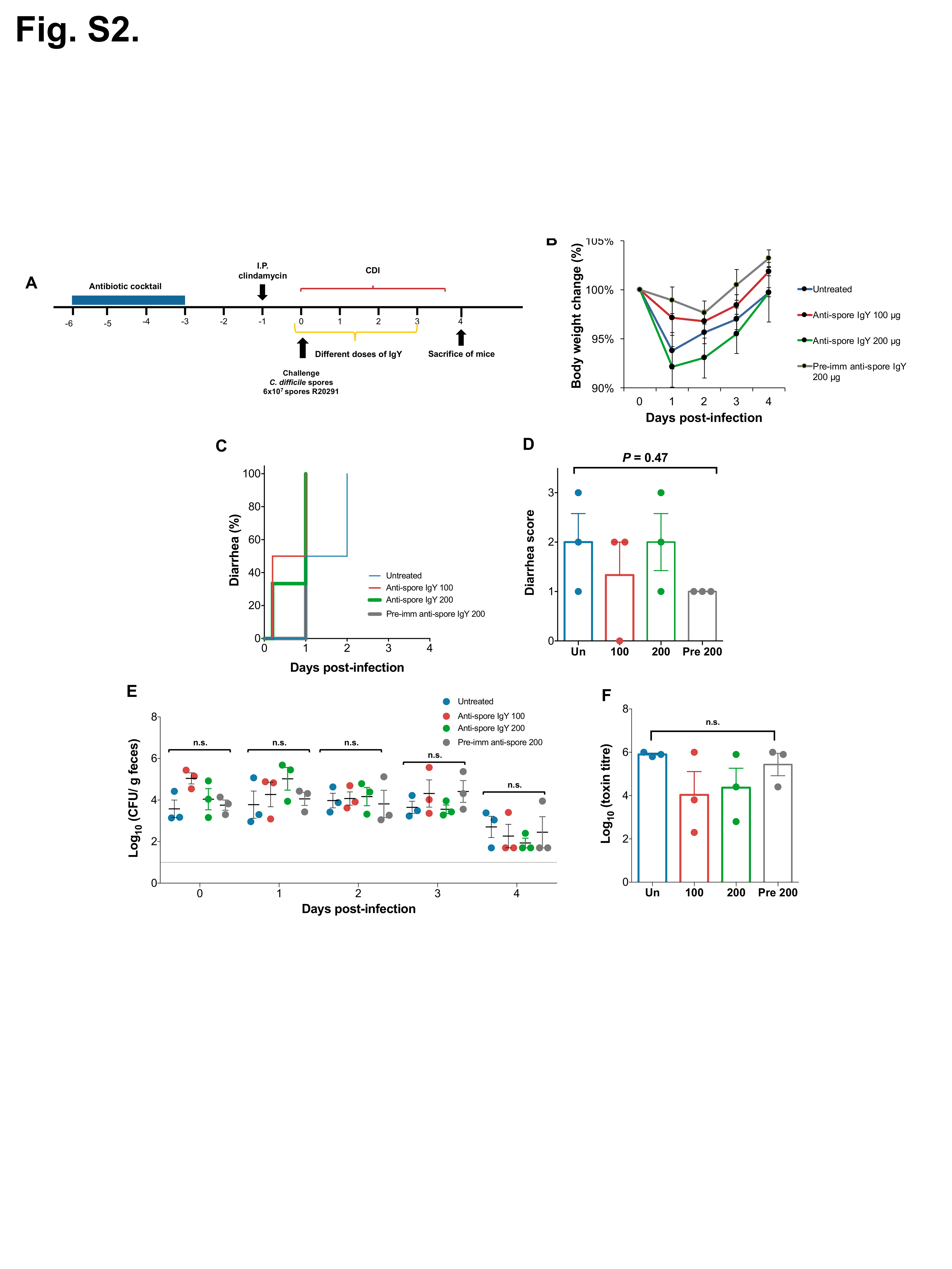

Supplement: Figure S2 — Administration of 100 and 200 μg of anti-spore IgY during the initiation of CDI. (A) Overview of the experimental design schematics for the prevention of initiation of C. difficile infection in a murine model. Antibiotic treated C57BL6 mice were infected with C. difficile R20291 spores (6 × 10^7^ spores; n = 3 per group) and subsequently treated with oral administration of anti-spore IgY or phosphate buffer saline as a control for 3 days. (B) Body weight change of untreated mice (blue line) and mice treated with 100 μg (red line) and 200 μg (green line) of anti-spore IgY and with 200 μg of pre-immunized IgY (gray line). C. difficile-challenged mice were monitored for: (C) time to diarrhea; (D) score of diarrhea; (E) Fecal C. difficile spore shedding; (F) Cecum content cytotoxicity at sacrifice; Data are representative of two independent experiments. Error bars are standard error of the mean. n.s., no significance; *P ≤ 0.05; **P ≤ 0.01. (D,F) Un, untreated; 100, is 100 μg of anti-spore IgY; 200, is 200 μg of anti-spore; Pre 200, is 200 μg of pre-immunized anti-spore IgY. [file Image2.TIFF]

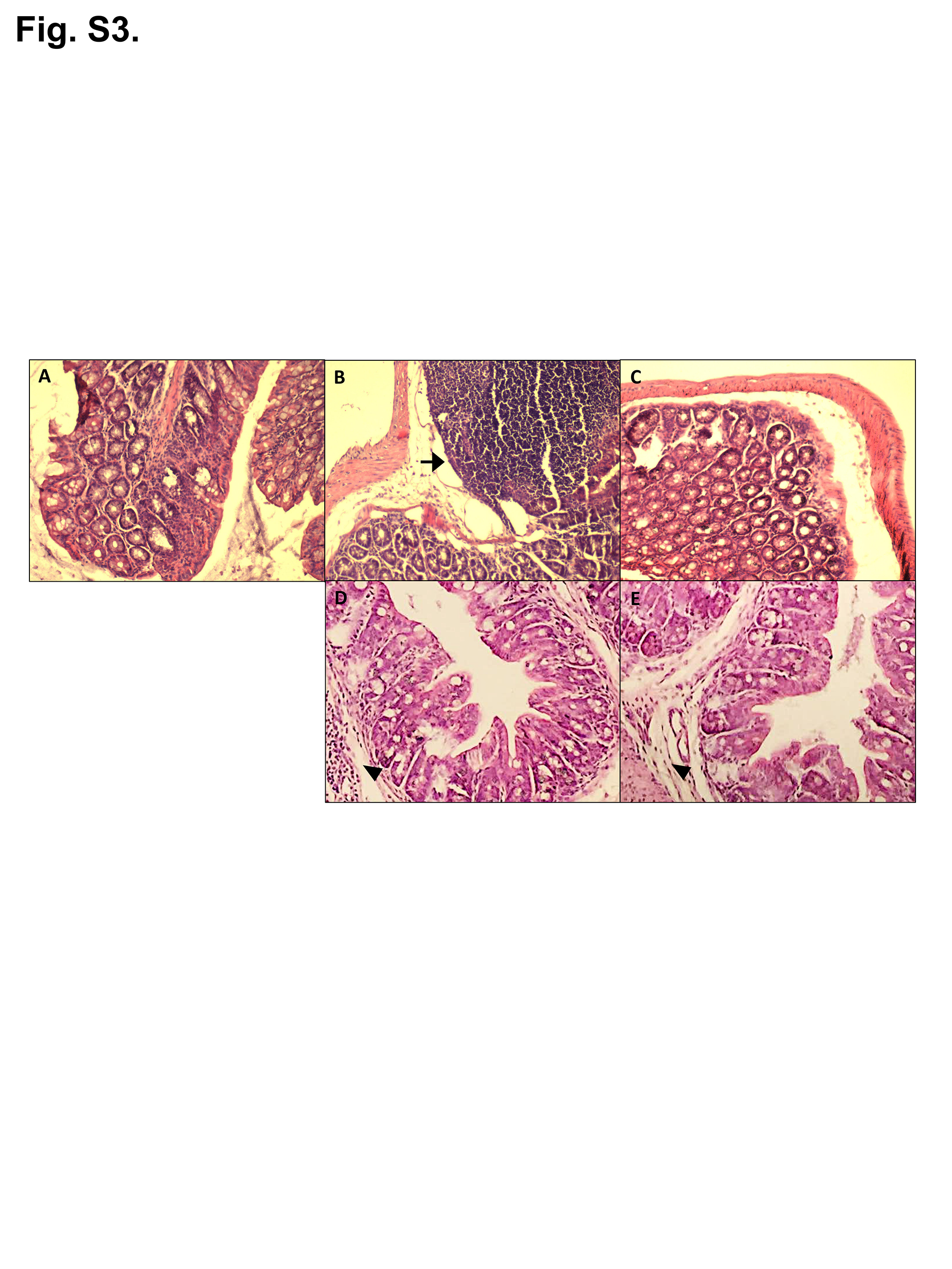

Supplement: Figure S3 — Histological examination of colonic tissues obtained from antibiotic treated mice exposed to C. difficile. Micrographs show colonic tissue of: (A) Non-infected mouse colon; (B) Colon from mice infected with C. difficile (control in initiation model); (C) Colon from mouse treated with IgY anti-spore prior and after infection (initiation model); (D) Colon from mouse infected and treated with vancomycin (recurrence model); (E) Colon from mouse infected and treated with vancomycin combined with IgY anti-spore. Black arrow shows edema, and arrow head shows neutrophilic recruitment. [file Image3.TIFF]

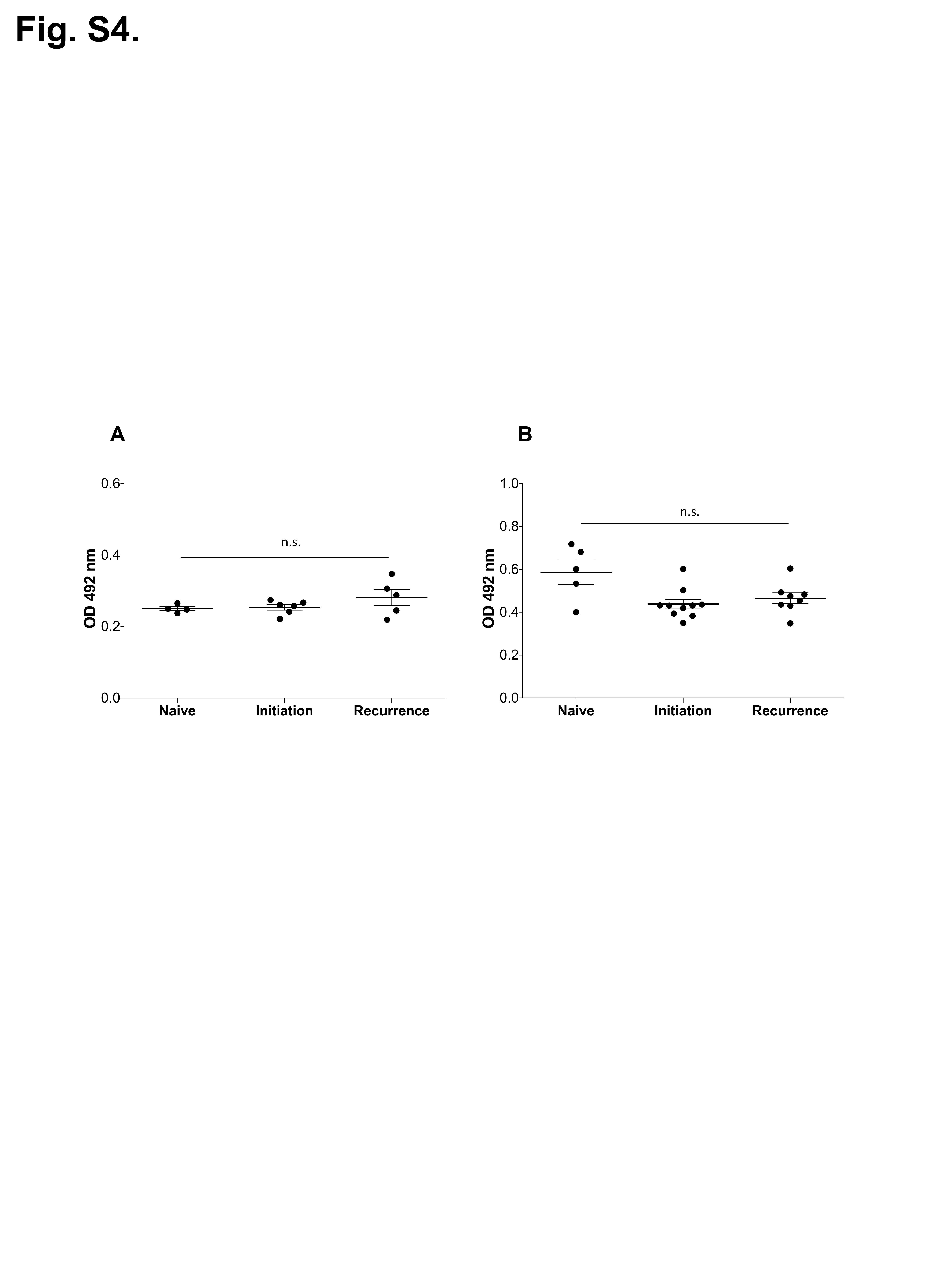

Supplement: Figure S4 — Serum titers raised against IgY and C. difficile spores during initiation and recurrence of CDI. Serum of naive mice, as a control, mice of the infection experiment (infection), and mice of the recurrent infection experiment (recurrence) were isolated and reactivity against C. difficile spores (A) and anti-spore IgY (B) was analyzed by ELISA as described in Methods. [file Image4.TIFF]
